# Healing Hands: The Tactile Internet in Future Tele-Healthcare

**DOI:** 10.3390/s22041404

**Published:** 2022-02-11

**Authors:** Stefan Senk, Marian Ulbricht, Ievgenii Tsokalo, Justus Rischke, Shu-Chen Li, Stefanie Speidel, Giang T. Nguyen, Patrick Seeling, Frank H. P. Fitzek

**Affiliations:** 1 Centre for Tactile Internet with Human-in-the-Loop (CeTI), Technische Universität Dresden, Deutsche Telekom Chair of Communication Network, 01062 Dresden, Germany; stefan.senk@tu-dresden.de (S.S.); marian.ulbricht@mailbox.tu-dresden.de (M.U.); justus.rischke@tu-dresden.de (J.R.); frank.fitzek@tu-dresden.de (F.H.P.F.); 2 Mimetik GmbH, 01219 Dresden, Germany; ievgenii.tsokalo@mimetik.de; 3 Centre for Tactile Internet with Human-in-the-Loop (CeTI), Faculty of Psychology, Technische Universität Dresden, 01062 Dresden, Germany; shu-chen.li@tu-dresden.de; 4 Centre for Tactile Internet with Human-in-the-Loop (CeTI), National Center for Tumor Diseases, Technische Universität Dresden, 01062 Dresden, Germany; stefanie.speidel@nct-dresden.de; 5 Centre for Tactile Internet with Human-in-the-Loop (CeTI), Technische Universität Dresden, Chair of Haptic Communication Systems, 01062 Dresden, Germany; giang.nguyen@tu-dresden.de; 6 Department of Computer Science, Central Michigan University, Mount Pleasant, MI 48859, USA

**Keywords:** tactile internet, tele-healthcare, human-in-the-loop, multi-modal, multisensory perception

## Abstract

In the early 2020s, the coronavirus pandemic brought the notion of remotely connected care to the general population across the globe. Oftentimes, the timely provisioning of access to and the implementation of affordable care are drivers behind tele-healthcare initiatives. Tele-healthcare has already garnered significant momentum in research and implementations in the years preceding the worldwide challenge of 2020, supported by the emerging capabilities of communication networks. The Tactile Internet (TI) with human-in-the-loop is one of those developments, leading to the democratization of skills and expertise that will significantly impact the long-term developments of the provisioning of care. However, significant challenges remain that require today’s communication networks to adapt to support the ultra-low latency required. The resulting latency challenge necessitates trans-disciplinary research efforts combining psychophysiological as well as technological solutions to achieve one millisecond and below round-trip times. The objective of this paper is to provide an overview of the benefits enabled by solving this network latency reduction challenge by employing state-of-the-art Time-Sensitive Networking (TSN) devices in a testbed, realizing the service differentiation required for the multi-modal human-machine interface. With completely new types of services and use cases resulting from the TI, we describe the potential impacts on remote surgery and remote rehabilitation as examples, with a focus on the future of tele-healthcare in rural settings.

## 1. Introduction

The COVID-19 pandemic of the early 2020s has brought a renewed focus on tele-healthcare, especially to the general public, who have had no prior exposure to the topic. While focusing to a large degree on virtual doctor visits in the early stages of the pandemic, the broad adoption of these basic remote services provides an infliction point for the further adoption of tele-healthcare services. The demonstrated safe and effective use on a massive scale will result in reduced adoption hurdles in the long term, especially those based predominantly on misconceptions [1]. Similarly, the previously provider-oriented use that favored urban settings or large provider environments will increasingly permeate across specialty disciplines and medical practice sizes [2]. In turn, this trend should also alleviate long-term costs that are of concern, especially in rural areas [3]. As mentioned in [4,5], tele-healthcare services used by medical providers and their patients oftentimes are employed to fill gaps in the existing system. These gaps include provider shortages, especially in under-served geographical areas, and identified solutions include technology moving into the consumer market. Tele-rehabilitation, for example, has been the subject of multiple decades of research and implementations ranging from medical to personal exercise and leisure domains. Earlier in the 20th century, initial rehabilitation at home was driven by remotely-controlled haptic interfaces [6]. Similarly, low-cost remote Electrocardiogram (ECG) monitoring was prototyped as well [7]. The continued development in the following decades has led to more integrated systems in tele-rehabilitation and other domains, such as [8]. The need to provide remote real-time healthcare services that go beyond foundational patient monitoring is rapidly increasing with continued specializations and geographic distribution disparities of medical care providers. For example, assisting technologies increasingly rely on smart environments, such as the Internet of Things (IoT). One example is the support of visually impaired people to increase their mobility [9]. A broad overview of IoT approaches and their application for COVID-19 scenarios is provided in [10]. The authors describe several potential inroads for the integration of sensing devices as well as potential uses for robots in different settings. Similarly, in [11], the authors describe several different robotic technologies and approaches from a practical point of view. Their comprehensive survey describes robot-supported COVID-19 mitigation efforts for healthcare provisioning, including general tele-healthcare and tele-surgery scenarios.

Here, we consider surgeries as one particular example with significant potential positive impacts that can be realized independent of the COVID-19 scenario. Postoperative complications are amongst the leading causes of death worldwide [12]. According to recent studies, around nine million major complications occur each year based on an estimated 300 million surgeries worldwide [13]. According to several studies, there is evidence that a surgeon’s lack of individual expertise, as well as poor surgical skill, cause severe complications in patients [14,15]. New technological innovations in the operating room, such as robotic systems, might provide a novel way to tackle this problem. However, robot-assisted surgery has not lived up to its full potential and does not exceed the capabilities of mechanical solutions so far. We envision an improvement in patient care and outcome if such systems enable access to expert knowledge in order to facilitate intraoperative decision making or remote surgery applications [16]. In this context, Tactile Internet (TI) methods and technologies have the potential to pave the way for next-generation robotic surgery [17].

These overall developments indicate an increase in the demand of tele-rehabilitation and tele-surgery services, especially in rural areas that have limited human resources. Advanced services require human-in-the-loop, and stagnation of tele-healthcare advancements originated mainly from the degraded network performance in terms of high latency and inflexible in-network services. For example, the authors of [10] explicitly identified control stability as one implementation hurdle for tele-robotics in healthcare. That motivates further research towards a Tactile Internet with Human-in-the-Loop (TaHiL) [17]. The realization of the TaHiL possibilities requires an interdisciplinary approach due to the psychophysiological nature of human perception, action, and cognition at its core. Striving only for technological advancement will be time-consuming and cost-inefficient since the advancements might not be sufficient or humans cannot perceive the advancements.

Subsequently, this paper goes beyond merely summarizing the use cases and requirements of current tele-healthcare. This paper focuses on the HiL aspect and analyzes the latency requirements from psychology perspectives. In turn, the paper pushes the requirements down to future communication networks toward a Tactile Internet–a crucial element to enable tele-healthcare. Finally, this paper explores and outlines the potential benefits of TI in significantly increasing tele-healthcare coverage using actual geographical data.

The remainder of this article is structured as follows. In the following Section 2, we describe the TI’s challenges and limitations for both psychophysiological and technical considerations. We subsequently describe how the TI with humans-in-the-loop can be applied in telerobotic applications as well as tele-rehabilitation scenarios, each with its unique challenges. We provide a measurement example for an implementation of multi-modal data streams in Section 4 showcasing how network softwarization in upcoming 5G networks can be employed to realize this new paradigm before we discuss potential implications in Section 5 and conclude in Section 6.

## 2. The Tactile Internet

**Figure 1:**
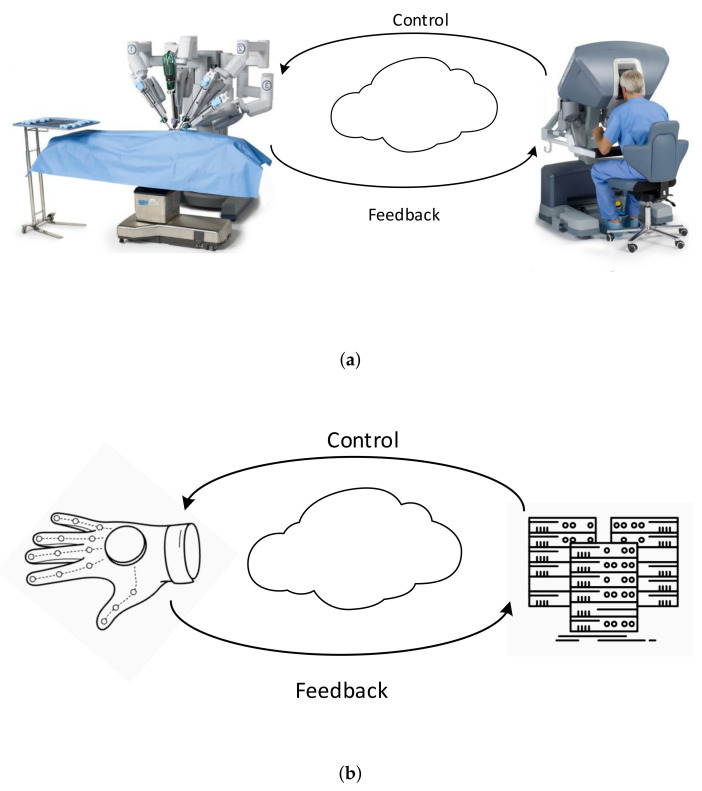
Examples for Human-in-the-Loop (HiL) control loops illustrating human–machine cohabitation principles. Human operators performing surgeries by remotely controlling a surgical robot and human patients wearing sensor gloves in tele-rehabilitation providing data interpreted remotely resulting in real-time treatment adaptation. (**a**) Human operators remotely controlling machines and receiving feedback. (**b**) Machines controlling human actions and receiving feedback.

Since the concept was introduced in 2014 [18], the TI research community has been advancing towards providing solutions that are highly applicable to the tele-healthcare use cases. At the Centre for Tactile Internet with Human-in-the-Loop (CeTI), we are working beyond the pure communication aspect of TI and focus on the HiL aspects. These consider the human multisensory perception and action as well as smart textiles with sensors and actuators to foster co-habitation between humans and machines. They also require new paradigms based on flexible, energy-efficient, and fast electronics [17]. Such research is trans-disciplinary: The aim of CeTI is to democratize access to skills and expertise to promote equity for people of different genders, ages, cultural backgrounds, or physical limitations. Tele-healthcare is one of the main use cases for the TI, which additionally includes industrial application scenarios and the Internet of Skills [19]. The latter is based on human–machine cooperation and indicates the need for transdisciplinary solutions. We include some commonly required round-trip times (including all sensing/actuating and processing) in Table 1. For tele-healthcare, where human actors are commonly placed within control loops, several approaches are now available to reduce the overall latency by tackling the individual components, as illustrated in Figure 1. We now describe the underlying requirements in greater detail.

### 2.1. Latency Requirements

The interconnected nature of healthcare-related services with a plethora of options and their interconnection is one of the most socially pressing concerns for tele-healthcare in general. However, such challenges can readily be solved through better cooperation of related software implementations at providers. Patient-centered results for tele-healthcare typically are reduced by a lack of direct physical contact options between healthcare professionals and their patients. That represents the main hurdle for tele-healthcare. In turn, the success of tele-healthcare essentially depends on the exchange of multi-modal signals between the medical professional and the patient. That requires suitable sensors and actuators on both sides, as well as secure and reliable real-time communication between both parties. We note that such digitally transmitted remote human–human and human–machine interactions rely on multisensory perceptual processing [23]. Thus, new technological developments need to consider psychophysical principles [24,25] and neurocognitive mechanisms [26] of human perception and action as well as how these may differ between persons of different ages [20], affected by brain development [27] or brain aging [28,29].

For tele-healthcare services that involve human–human or human–machine interactions with multi-modal signals, the latencies of current technologies at multiple levels are relatively slow. Even ignoring the network communication delay (at least on the order of 100 ms), most current device-to-device delays are slower than the information processing speed of neurons. In the brain, neurons in the somatosensory cortex, which supports tactile and haptic perceptual processing, operate with a temporal precision at the level of 1 ms. Although not as fast as the tactile modality, neurons in the auditory or visual cortex still operate with latencies that are faster than most technical devices, i.e., on the order of about 3 ms and 15 ms, respectively [21]. Such high speeds of human sensory processing (as well as inherent differences in the temporal requirements of the senses) pose challenges for designing sensors, actuators, and digital data coding and compression schemes that could provide coherent, naturalistic multi-modal feedback for tele-healthcare applications.

An additional challenge for developing multi-modal human–machine interfaces that could serve broad populations is the need to consider users of different ages. Empirical findings from lifespan psychology and cognitive neuroscience show that perceptual and cognitive processes change considerably across the human life span (see [20] for review). Evidence from empirical studies shows that when making perceptual decisions, children and old adults have lower levels of processing speed and processing robustness (i.e., higher degrees of random processing fluctuations) than young adults [30]. Parallel to such age-related differences in information processing speed and robustness, the temporal precision of neuronal signals is also lower in children and in older adults, relative to younger adults [31]. Taken together, age-related differences in multisensory perception and action challenge the one-size-fits-all assumption in technical designs. They will likely break usability for users at the two ends of the lifespan if age-sensitive parameters of key human factors are not considered en route to new technological developments.

### 2.2. Current Latency Limitations

**Figure 2:**
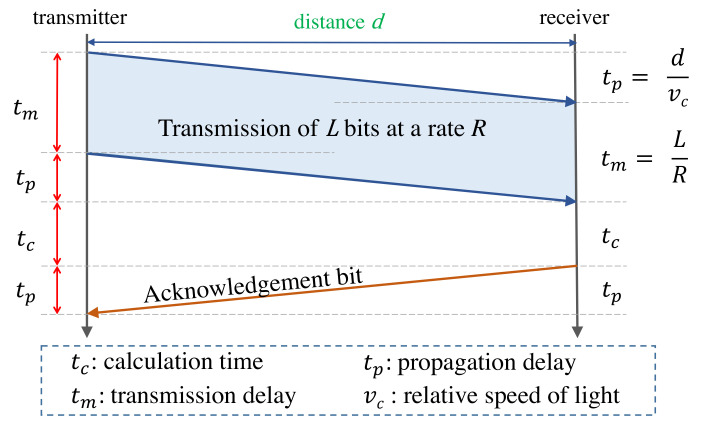
Breakdown of the total round-trip latency into the different latency components $t_{p}$, $t_{m}$, and $t_{c}$, when a transmitter communicates with a receiver.

Safe and reliable real-time communication (or, more precisely, human-perceived real-time communication) can only be realized with sufficient bandwidth and low latency. When designing a communication network, the desired latency has to be considered from the beginning and, in contrast to the bandwidth, cannot or can hardly be optimized later. The overall experienced latency is not a singular quantity, however it is composed of several components. Figure 2 illustrates an example of communication between a transmitter and receiver with different latency components. The message of the size *L* bits shall be transmitted over a distance *d* with the data rate *R* in bits per second and later, after a processing time for computation $t_{c}$, shall be confirmed by a one-bit acknowledgment response. The total time to transmit the message of *L* bits at a rate of *R* bits per second is $t_{m}=L/R$. As it takes a certain amount of time after the transmitter has sent the first bit until this bit actually arrives at the receiver (and vice versa), we denote this propagation delay as $t_{p}$. Therefore, the total round-trip delay is:

(1) $$T=2*t_{p}+t_{c}+t_{m}.$$

The propagation delay $t_{p}$ as in Equation (1) and illustrated in Figure 2 generally depends on the distance between transmitter and receiver and the type of transmission medium. One could further detail $t_{p}$ (in case the transmission path consists of several intermediate nodes) as in Equation (2):

(2) $$t_{p}\left(N,t\right)=\sum_{n}t_{pl}\left(n\right)+t_{pn}\left(n,t\right).$$

In Equation (2), the overall delay is composed of the individual transmission line segment’s physical signal propagation time $t_{pl}\left(n\right)$ and a node-specific processing delay $t_{pn}$. Both are aggregated over the total number of network nodes *N* connecting the individual segments, with n=1,…,N. For an individual segment *n*, $t_{pl}\left(n\right)=d\left(n\right)/v_{c}\left(n\right)$ depends on the segment’s distance d(n) and the relative speed of light signals propagate through the segment, $v_{c}\left(n\right)=f\left(c,n\right)$, whereby *c* denotes the speed of light *c*. Omitting the node-specific notation for clarity, $v_{c}$ is assumed to be about 66% of *c* if copper cabling is used and nearly 100% of *c* if a fiber-optic cable is used. In addition to the distance and medium-specific propagation delay components, $t_{p}$ with intermediate nodes also includes a node-specific delay, $t_{pn}\left(n,t\right)$. This delay component consists of a packet processing and storage part $t_{pp}\left(n,t\right)$ as well as a component caused by the queuing and interaction with other packets of the network $t_{q}\left(n,t\right)$, i.e.,

(3) $$t_{pn}\left(n,t\right)=t_{pp}\left(n,t\right)+t_{q}\left(n,t\right).$$

Jointly, we derive Equation (4) for a fine-grained view of $t_{p}$ as:

(4) $$t_{p}\left(N,t\right)=\sum_{n}\frac{d(n)}{v_{c}\left(n\right)}+t_{pp}\left(n,t\right)+t_{q}\left(n,t\right).$$

We drop the identifiers for node and time in the remainder of this contribution for clarity.

As described in Equation (4), the total propagation delay is caused by the combined packet delays at the individual network nodes $t_{pn}$ and, in turn, correlates with the number of nodes and their respective traffic load. The traffic load can be considered as an M/M/1 system. In such a system, the meantime in which a packet will have to wait for transmission is detailed as:

(5) $$t_{q}=\frac{1}{\mu -\lambda }-\frac{1}{\mu }.$$

In Equation (5), the delay is constrained by the line utilization, which refers to the ratio of the maximum line rate μ and the packet arriving rate λ. If the line is utilized more than 80%, the packet waiting time rises significantly. However, the statistical approach for mean queuing delay optimization is not applicable if important and delay-critical data is transferred through the network. Therefore, we consider measurements of methods providing hard real-time conditions for a node’s queuing delay for our experiments described in Section 4.

For the calculation time $t_{c}$, we would generally assume very low values. As this is an active research and application domain, continuous development is taking place to combine the different participating entities [32] and to reduce the burden of developing applications in this environment [33]. Even if we considered multiple inputs from several sensors that need to be evaluated, such computation would not need considerable time in software on bare hardware. However, virtualization is required to implement the multi-access edge cloud as described beforehand. Virtualization is the key to the new communication networks and reducing the propagation delay. On the other hand, virtualization can increase the calculation time $t_{c}$. In previous work [34], this was detailed together with approaches to reduce this effect without completely eliminating it.

Equation (1) provides a framework to analyze the impact of the distance between a transmitter and receiver on the total round-trip time that has to meet the rigid constraints of real-time applications, such as tele-healthcare. In the following, we further analyze the dependency by a motivating example. In order to receive a message of length *L*, the receiver listens for the time $t_{m}$, the transmission time, and evaluates the message for computing results during the computation time $t_{c}$. After the calculation, the receiver sends a message with the result back to the transmitter, corresponding to the propagation delay. If the acknowledgment is composed out of more than one bit, we should proceed as we did with $t_{m}$ beforehand. High data rates can minimize the transmission time $t_{m}$ relatively easily. For example, $t_{m}$ is roughly 32 ns for a message of the size 40 bytes, which is typical in robotic applications, on a 10 Gbit/s link. Therefore, a further reduction of $t_{m}$ does not constitute an overall significant problem.

As explained before, the propagation delay for a line segment $t_{pl}$ depends only on the distance *d*. The light covers a distance of 300 km per ms. For tele-healthcare applications between America and Europe with, e.g., a 9000-km distance, this would already be 30 ms and, thus, be too large for real-time applications. To enable tele-healthcare applications between greater distances, models also referred to as digital twins [35] are created from the operator or the patient and held as virtual objects in the communication network itself, in the so-called multi-access edge cloud. These virtual objects can then be calculated in the vicinity of the respective counterparts, thus *virtually* reducing the distance *d*. We currently assume that the maximum distance to a multi-access edge cloud is 25 km, which in turn would limit $t_{p}$ to 1/12 of a ms.

This section analyzes the most critical metric of the Tactile Internet: latency. Future communication networks have to strive toward even lower latency limitations to realize the TI. That allows for expanding the geographic coverage area for novel services, which we will discuss in Section 5. Simultaneously, the Tactile Internet needs to enable innovative solutions, such as digital twins, to realize expansions beyond physical limitations. The following section will explore two potential use cases that can significantly benefit from the Tactile Internet.

## 3. Tele-Healthcare Latency in the Real World

We now shift the point of view to demonstrate some of the intricacies and new approaches to tele-healthcare services in these forthcoming networks.

### 3.1. Surgery

#### 3.1.1. Remote Surgery

Surgery is one of the most difficult psychomotor activities, and a certain amount of first-hand experience is necessary to obtain proficiency, e.g., for colorectal cancer surgeries, 60–80 operations are needed [36]. This number will increase even more due to the steady introduction of new techniques, devices, and robotic systems [37]. Remote surgery refers to the ability to perform surgical interventions over long distances between clinician and patient. The surgeon usually sits on a console and controls a telemanipulated robotic system in a physically distanced operating room via a high-speed connection. That offers the opportunity to get access to expert skills on a global scale while conducting complex interventions, e.g., cancer surgeries where the outcome depends considerably on the surgical experience [15]. In such scenarios, it is entirely conceivable that the expert performs the most critical and complex phases instead of the whole surgery.

A popular example in a clinical routine that enables remote surgery is the da Vinci^®^ Surgical System. Although it has been proven that telesurgery is feasible [38], it is still not common due to different challenges, e.g., regarding communication infrastructures, such as latency, reliability, and security [39,40]. In addition, surgical interventions would also hugely benefit if there would be a possibility for telementoring, which would be applicable to not only robotic procedures [41,42,43,44].

#### 3.1.2. Intuitive Human–Machine Coworking

Human-in-the-Loop applications in surgery based on Tactile Internet technologies go far beyond remote surgery and telementoring. Future surgical robotic systems will exceed the capabilities of telemanipulation by enabling a context-aware human–machine collaboration, e.g., by providing the right assistance at the right time, similar to an experienced human assistant [45]. Therefore, the system has to understand a given situation, act on it, and learn from it. Examples of context-aware assistance during surgery range from visualization of risk and target structures to semi-automation of specific tasks on a robotic platform, e.g., suturing, laparoscope guidance, or sonography tasks [16].

For enabling context-aware assistance and transfer of skill to robotic platforms, surgical skills need to be captured based on expert demonstrations in sensor-enhanced environments, such as an operating room [46,47]. Multi-sensor acquisition leads to a vast amount of sensor data that require efficient solutions for data compression, especially for haptic data [48]. Machine-learning approaches allow to model surgical skills based on annotated sensor data [16,49] and allow semi-automation of surgical skills such as knot-tying, suturing, laparoscope guidance, or sonography tasks [50,51,52,53].

In order to enable real-time human–machine interaction during surgery, the context and progress of the surgical workflow have to be automatically and continuously perceived, analyzed, and predicted [54,55]. To complement the machine-learning-based approaches to achieve this goal, novel reasoning techniques that compete with latency constraints and feedback requirements and mimic human decision-making have to be exploited. Overall, such methods could enhance the collaboration between surgeons and cyber-physical systems, democratize surgical skills by quantifying surgical experience, and make it accessible to machines.

### 3.2. Rehabilitation

#### 3.2.1. Independent Rehabilitation at Home

Stroke continues to be a leading cause of long-term disability in the United States (U.S.) [56] and functional recovery varies greatly based on several socio-economic factors. According to [57], discharges to home health care rather than skilled nursing facilities in the U.S. yields cost savings, however outcomes should be improved. One rehabilitation trend, especially for stroke survivors, is the use of technology to augment the long-term recovery treatment plan for patients. Electro-mechanical and robotic gait training assistance can increase the possibility of independent walking [58,59] with competitive costs [60]. The broad availability of immersive Virtual Reality (VR) devices and inputs adopted for rehabilitation has found broad adoption and good results [61], especially when specifically tailored [62] or with gaming elements [63]. Examples for involving recovering patients in immersive environments that include capturing motoric interactions include wearable devices, such as gloves [64,65]. Typically, design considerations for wearable gloves in this context require soft materials, which makes these gloves suitable for comprehensive, long-term treatment across underlying causes [66,67,68,69]. Similarly, the combination of virtual reality environments and wearable gloves can be used to determine the hand motor performance for active daily living activities [70].

#### 3.2.2. Intuitive Machine-Guided Human Rehabilitation

Future trends will require even more modalities in feedback loops that can move into the at-home treatment domain. For example, the increase in Alzheimer and dementia patients will require new modalities of treatment to prolong independent living, such as combinations of VR and brain stimuli [71]. Overall, the trend to more wearable devices will hasten as current bottlenecks are resolved [72]. This prominently places the human patient in the control loop, providing feedback through sensors. The interpretation of the feedback can be by caregivers in real-time or be fully automated, requiring the ultra-low latencies of the TI.

Consider a scenario where remote patient connectivity is required to combine real-time care while removing the need to travel to remote locations, effectively increasing the number of patients that could directly be treated, even with human caregivers. Combining the different needs for the interaction of the tactile, auditory, and visual senses poses three different challenges with respect to bandwidth and latency, additionally limiting the geographical distance that can be covered unless special efforts are taken [35]. For example, the Cyberglove-II from *CyberGlove Systems* (http://www.cyberglovesystems.com, accessed on 29 November 2021) employs 22 sensors and generates 1 byte of measurement data per sensor every 10 ms, which places the time resolution below the 1-ms threshold needed to provide fully immersive real-time experiences. Wearable devices currently under development by *Mimetik* (https://www.mimetik.com, accessed on 29 November 2021) generate similarly frequent data every 10 ms, however with finer granularity and more information, which results in 240 bytes for each measurement. In the best-case scenario without any intermediate processing (i.e., direct projection of sensing/actuating between two human partners over the network), this would limit the range to about 25 km for network traffic round-trip latencies within the 1-ms boundary.

In summary, tele-healthcare services can significantly benefit from the Tactile Internet. However, tele-healthcare use cases typically require the transmission of different data streams for multiple modalities, such as audio, video, and haptic signals. Each has drastically different traffic characteristics, such as bandwidth and reliability requirements. Furthermore, human sensitivity is remarkably different when interacting with different modalities. That poses another critical challenge for the underlying communication networks, which need to differentiate different traffic types and apply different priorities. We will further explore this challenge in the next section.

## 4. Latency and the Problem of Net-Neutrality

Closing the loop of this contribution in this section, we again note that the realization of advanced tele-healthcare services requires significant underlying communication network capabilities. Identified in [10], control stability–here provided through the Tactile Internet with Human-in-the-Loop–is one requirement for the adoption of advanced tele-healthcare services. While typical installations of communication networks today exhibit several limitations, enhancements that will increase the network capabilities are at the cusp of mainstream deployment. In the remainder of this section, we provide a first view on the capabilities of devices in these deployments to realize the TaHIL and, subsequently, remove the control loop obstacles for implementation of advanced tele-healthcare services.

As the multi-modal communication between a human operator and a remotely controlled device (e.g., as a robot) is composed of several components, such as audio-visual and tactile parts, these communication streams would compete with each other in a shared communication network with net neutrality. As described in Section 2.1, each sense has its own bandwidth and latency requirements. Net neutrality would ensure that all senses are treated equally, and no prioritization would be possible. This is based on the notion of Internet Packet transparency in the net neutrality concept, where no additional knowledge of content is available [73]. However, advances that enable per-stream handling of network data to allow the individual prioritization of different network streams, each with a dedicated Quality of Service (QoS) [74]. Jointly with the advances of virtualization throughout communication networks, this enables the possibility of splitting a physical communication network into parallel logical communication networks with different bandwidth and latency performance characteristics.

### 4.1. Evaluation Setup and Experimentation

We now consider an example for the applied separate managing of network traffic at an intermediate FibroLAN Falcon-RX/G switch through QoS provisioning with the MoonGen [75] traffic generator as follows. The testbed is based on commercial-off-the-shelf (COTS) hardware. Advances in Open RAN technology made COTS hardware increasingly more popular for research and implementation. Five nodes are employed in our setup to represent various traffic priorities: One node is used as sink whereas two nodes transmit cyclically (in the case of the *tactile* stream even isochronously) data, and the remaining two nodes behave as interfering network participants. The data streams pass through the device-under-test (DuT), i.e., the FibroLAN switch. In order to evaluate the packet processing characteristics of the switch, the background traffic is varied for Ethernet frame sizes and network load. Subsequently, we are able to derive $t_{pn}\left(n,t\right)$ as defined in Equation (3). Since two nodes are utilized with a data rate corresponding to *R* = 1 Gbit/s on all ports, an overload situation at the sink’s network port is created. By inserting accurate timestamps into the high-priority Ethernet frames, the residence time of the frames can be measured for different situations with an accuracy of nanoseconds. Furthermore, the measurement is intended to estimate the need for high-cost scheduling fabrics, e.g., *TSN*-capable hardware, or if it is still sufficient to use widely-existing IEEE 802.1Q technology regarding the different payload types and their requirements.

For the high-priority data streams, we consider three major types of network traffic representing *tactile*, *audio*, and *video* network slices. The assumed traffic employed for the slices was generated and prioritized without significant propagation delays. The *tactile* slice has the highest priority and is generated every millisecond following the IEEE P1918.1 Tactile Internet Working Group’s Haptic Codec Task Group reference data set, described in [76], as 82-byte User Datagram Protocol (UDP) traffic. The *audio* traffic has the second-highest priority and is generated as 480-byte UDP datagrams every 24 ms, while the *video* UDP traffic is generated from the highly dynamic *Big Buck Bunny* video traces from [77] with the lowest priority. We also generated background traffic, an Ethernet stream with a load of 120%, for over-saturating the link in order to force losses and frame drops.

### 4.2. Results

**Figure 3:**
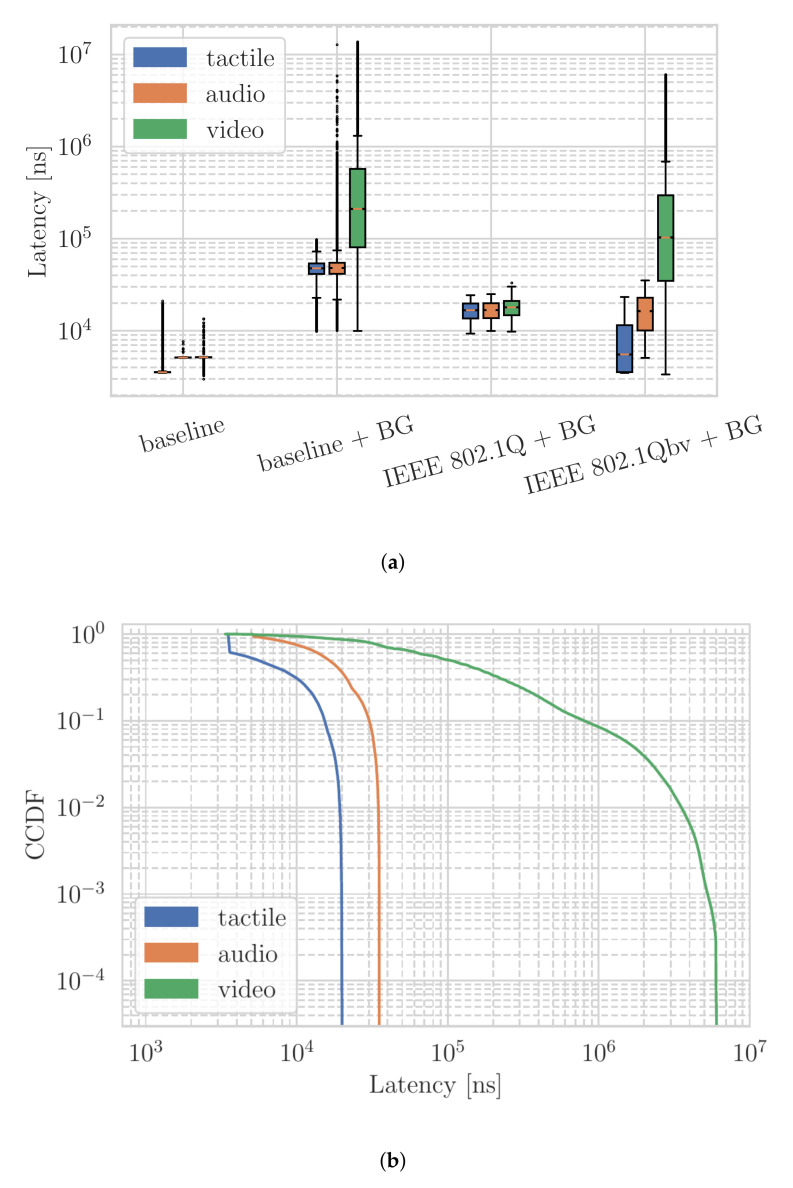
Example measurement results for the *tactile*, *audio*, and *video* network traffic, individually prioritized. Without background traffic, the latency is predominantly driven by the Ethernet layer frames’ sizes and counts. With competing background traffic, the network switch handling of additional frames results in overall latency increases. While traffic prioritization does not separate well three slices of traffic, which is an unexpected result, traffic prioritization and time-aware shaper can separate three slices of traffic and benefit the sensitive tactile traffic the most. (**a**) Latency of three slices of traffic in four measurement scenarios. (**b**) Complementary Cumulative Distribution Function (CCDF) of three slices of traffic when the time-aware shaper is applied.

In the following, we describe measurement results for four scenarios: (a) *baseline* with only the described tactile, audio, and video data streams; (b) *baseline + BG* (background) with the same three data streams with the addition of the background traffic stream; (c) *IEEE 802.1Q + BG* that enables strict priority QoS settings within scenario b; and (d) *IEEE 802.1Qbv + BG* which replicates scenario b with QoS settings and the time-aware shaper feature. The box plots illustrate the resulting latency of the three prioritized data streams in four scenarios in Figure 3a.

In the first measurement scenario (baseline), we initially observe that all three slices of network traffic are delineated. The majority of haptic traffic experiencing delays below 3 μs, while the latency of the audio stream and the significantly larger video stays around 6 μs. However, in scenario b with the background traffic, the median latency of the tactile and audio slices increases to almost 60 μs. In comparison, the median latency of the video stream increases to above 200 μs for haptic, audio, and video, respectively. In scenario c, when QoS is applied, all traffic slices’ latency was reduced significantly compared to scenario b when there is no QoS. However, the latency of all slices is still significantly higher than those in scenario a, i.e., without background traffic. In the last scenario, i.e., with traffic shaper, the latency of the audio stream is comparable to that of scenario c. Notably, the median latency of the tactile stream approaches that of scenario a–without background traffic. In contrast, the latency of the video stream increases the most to nearly 100 μs. It is worth noting that IEEE 802.1Qbv with a time-aware shaper can distinctively separate three traffic slices. We further illustrate in Figure 3b the latency distribution of the three slices in scenario c as a Complementary Cumulative Distribution Function (CCDF).

With the extremely fine-grained requirements for measurements in our setup, it is additionally useful to estimate the quality of the measurement results. We consider the confidence interval [78]. Employing the *tactile* data stream in the *baseline* measurement as an example, we note that the experiment results in *n* = 550.503 samples with a mean delay of μ=3584.105 ns and a standard deviation of σ=548.780 ns. Subsequently, at the α = 95% confidence interval level for the observed mean delay at the switch, one would derive $\mu_{\alpha =0.95}=3584.105\;ns\pm e$, where the absolute error is $e_{\alpha =0.95}=1.450\;ns$. Differently put, one can be highly certain that the actual mean value will fall within a few nanoseconds *e* of the determined average. Note that we consider latency limits of around 0.5 ms as one threshold as in Table 1, for which the potential error is still several magnitudes smaller.

Table 2 provides an overview of the determined absolute errors *e*. The rather high values for the fourth measurement case can be explained by closed queue gates at the DuT. Since the transmitting nodes and the DuT are not synchronized (as opposed to IEEE 802.1Qch), the packet delay variation increases. It is essential to understand that although the jitter increases, the overall latency is bound and lower compared to the measurement case *IEEE 802.1Q + BG*.

Provided the highly demanding and ultra-low latency requirements for this tactile traffic to enable processing, any reduction of the in-network delay can yield significant benefits for applications and services.

## 5. Discussion

The Tactile Internet provides the framework for the future democratization of skills and expertise, with significant impacts on tele-healthcare. The vast potentials for augmentation of care delivery in its various aspects are manifold and could change the manner of delivering care to commonly under-served populations as well as provide completely new means of care delivery. The suburban and rural communities surrounding smart cities commonly do not have the same level of services, which oftentimes can be attributed to the different demographics as well as financial wellness of these communities. This, however, is in stark contrast to the needs of these communities. For example, the average population fraction over 65 in rural communities in the United States currently is estimated around 17.5%, while that for urban areas is 13.8% [79]. In turn, the needs for services provided, especially in the healthcare domain, can be assumed higher in rural areas, albeit services are less available.

As an example of the potential reach that upgraded tele-healthcare could provide, we exemplary consider the spread of hospitals for a rural area in Michigan, USA. Data for hospitals and cell phone towers were obtained from [80,81] and subsequently processed as follows. For each listed hospital, we evaluate the closest cellular towers within a 15-km distance to determine the potential for the hospital to reach beyond its current capabilities and remain within requirements for a 1-ms TI service. On average, we determine a distance of 8.8 km (standard deviation of 4.08 km) from a hospital to the nearest cellular towers within a 15-km radius for the entire United States.

**Figure 4:**
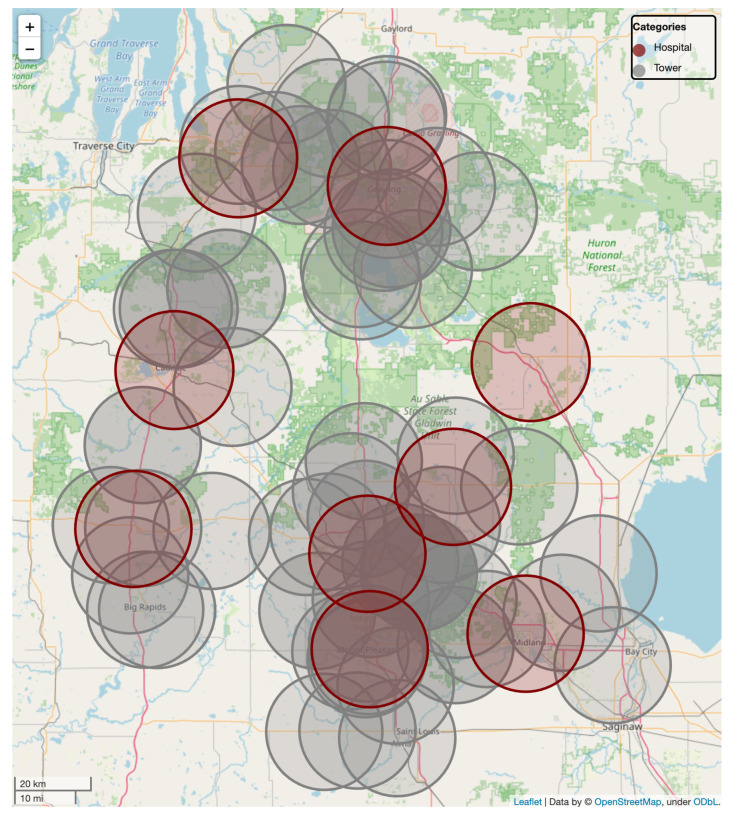
Example scenario for the increased reach of tele-healthcare in rural communities. While this example area in central Michigan is surrounded by hospitals, their individual reach (maroon) would only cover the closest areas, assuming a 15-km range to enable a 1-ms latency. Provided with nearby cellular towers, the majority of the region could be serviced as well within the latency requirements. Reducing the latency requirements incrementally would increase the coverage further.

For the area under consideration, we provide an illustration in Figure 4.

In the figure, we illustrate a general area in the northern center of the state, characterized by a main roadway in the center and fairly sparsely settled areas all around. Hospitals from the data set are indicated in maroon with their respective assumed range of 15 km around a hospital as well as each existing cellular tower, colored in grey. Even with this range limitation to enable the Tactile Internet’s capabilities, it becomes visible that a significantly larger range can be covered than just by providing networked services from a hospital itself. While some areas in the center of the figure clearly remain out of service, the overall area has shrunk significantly. Were the range of hospitals, distance to cellular towers, or tower range relaxed, it would be possible to cover the entire area where before, no remote medical service would be feasible at the level enabled by the TI. Instead, either caregivers would need to travel to patients, or patients would need to travel to distant caregivers, which is not always a feasible proposition. Overall, this illustrative example highlights the vast potential that lies in the Tactile Internet’s application for tele-healthcare scenarios, especially for rural areas that commonly are under-served.

## 6. Conclusions

The Tactile Internet and built-upon solutions such as tele-healthcare have tremendous opportunities to increase the overall quality of life. This effect can be greater for those in former under-served areas as well as provide a long-term incentive countering the globally experienced rural flight. As such, the Tactile Internet can be a significant catalyst for societal change. This paper provides the underlying requirements from both medical as well as technical high-level points of view and highlights hurdles and opportunities for adoption. Detailed measurement test beds that enable an end-to-end evaluation are currently rare, especially considering that full roll-outs of the actual networks and their capabilities have just begun. In future works, we intend to evaluate testing environments and conduct performance evaluations thereof.

## Figures and Tables

**Table 1:** Latency requirements for different types of interaction.

Interaction/Applications		Round-Trip Latency Requirement
pxHuman–Machine Interaction (Sensitivity to Neural Timing among Cortical Areas [20,21])	Tactile	<1 ms
	Auditory	<3 ms
	Visual	<15 ms
Motion Control Applications [22]	Machine Tool	<0.5 ms
	Packaging Machine	<1 ms
	Printing Machine	<2 ms

**Table 2:** Absolute error *e* in nanoseconds for the individual data stream measurements, as discussed in Section 4.2.

	Tactile	Auditory	Visual
*Baseline*	1.450	0.772	3.172
*Baseline + BG*	5.049	44.576	273.675
*IEEE 802.1Q + BG*	1.451	5.042	4.128
*IEEE 802.1Qbv + BG*	13.756	98.081	771.183

## Data Availability

The data presented in this study are available on request from the corresponding author.
